# Atypical presentation of tight filum terminale with thoracic disc herniation: a case report

**DOI:** 10.1186/s13256-024-04371-z

**Published:** 2024-02-04

**Authors:** Taiju Miyagami, Hidetoshi Nojiri, Satoru Okada, Kiichi Mitsumoto, Kosuke Uemura, Toshio Naito

**Affiliations:** 1 Department of General Medicine, Faculty of Medicine, Juntendo University, 2-1-1, Bunkyo-ku, Tokyo, 113-8421 Japan; 2 Departments of Orthopaedics, Faculty of Medicine, Juntendo University, Tokyo, Japan; 3 Department of Family & General Medicine, Tokyo-Kita Medical Center, Tokyo, Japan

**Keywords:** Tight filum terminale, Disc herniation, Upper limb pain, Back pain

## Abstract

**Background:**

Tight filum terminale is a rare and challenging condition to diagnose because it presents with nonspecific symptoms and unclear imaging findings. This report documents an atypical case of tight filum terminale.

**Case presentation:**

The patient was a previously healthy Asian 18-year-old male presenting with recurrent upper extremity and back pain, initially treated as nonspecific musculoskeletal pain. Notably, the patient’s symptoms were inconsistent with the dermatome, showing no correlation with his skin’s sensory innervation areas. In contrast to typical tight filum terminale presentations focused on lower extremity and lumbar region disturbances, this patient experienced pain and weakness predominantly in the upper extremities and back, hypothesized to result from traction myelopathy exacerbated by thoracic disc herniation. Investigations including blood and nerve function tests were inconclusive. However, a magnetic resonance imaging scan revealed a combination of tight filum terminale and tiny thoracic disc herniation. A diagnosis of tethered spinal cord syndrome was confirmed following further tests and imaging. The filum terminale was surgically removed, resolving the symptoms at a 7-month follow-up.

**Conclusions:**

This case underlines the importance of including tight filum terminale as a differential diagnosis in cases of unexplained upper or lower extremity pain. Primary care practitioners, particularly those managing undefined symptoms, should consider tight filum terminale in their diagnostic approach.

## Background

Tight filum terminale (TFT) is a type of tethered cord syndrome (TCS), in which the low spinal cone and myelomeningocele are absent [1]. Khoury *et al.* first reported TFT in 1990 [2]. Since then, TFT has been suggested to possibly occur in approximately 14–28% of patients with TCS [3]. In TFT, hypertonia of the filum terminale causes spinal cord traction, resulting in neurological symptoms inconsistent with the dermatome, as well as bladder and rectal disturbances [4]. In children, incontinence and frequent urination occur, with incontinence being particularly common. However, in adults, frequent urination is more common [4, 5]. This condition can occur at any age, including during growth, overloading the spinal cord during sports, and other activities or age-related degeneration [3]. TFT is a rare disease that is often difficult to diagnose. In this report, we describe a case of atypical presentation of TFT.

## Case presentation

A previously healthy Asian 18-year-old male patient presented to our hospital with recurrent pain in his upper extremities and back. He had no family history of note. A total of 2 years previously, he had experienced a minor soccer collision and a sudden subsequent onset of severe left-sided back pain and left upper limb muscle weakness. His symptoms improved within 4 weeks of receiving nonsteroidal anti-inflammatory drugs from his nearest clinic. In the 2 years that followed, symptoms had reappeared 10 times, but each episode resolved within a few weeks.

Vital signs were normal; however, physical examination revealed severe spontaneous pain in the left upper back and left side of the chest that was inconsistent with the dermatome. Weakness was also observed in the left trapezius muscle and the entire left upper extremity. Muscle strength was approximately 3 on the Medical Research Council grade scale. Additionally, there was a hyperesthesia in the proximal left upper extremity that did not conform to the dermatome. Deep tendon reflexes in both upper and lower limbs were unremarkable, and there were no rectal disturbances.

**Fig. 1:**
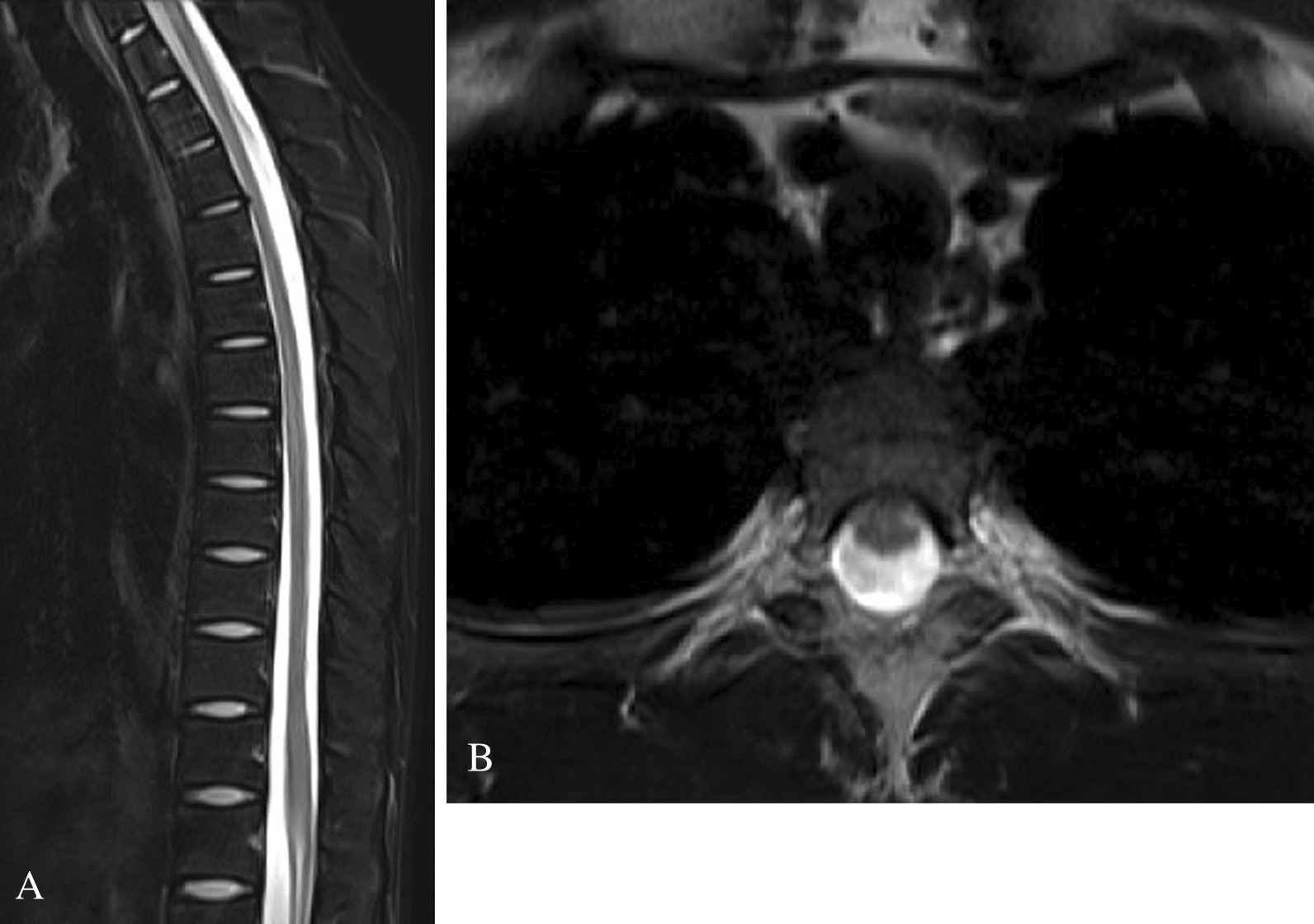
Magnetic resonance imaging (MRI) findings of the cervical and thoracic spine. (**a**) Sagittal MRI (T2-weighted) of the thoracic spine with tiny herniation of Th4–5 and passage of the spinal cord through the upper and lower vertebrae at the shortest distance. (**b**) Transverse MRI (T2-weighted) shows decreased spinal fluid in the anterior spinal cord in the Th4–5 region

Blood tests were performed for adrenal insufficiency, hypothyroidism, and electrolyte and trace element effects; however, no abnormalities were found. Under the suspicion of thoracic outlet syndrome, Adson and Wright tests were performed, both of which yielded negative results. Although the symptoms were not in line with the dermatome, this patient was considered to have back muscle or spinal cord dysfunction, and thus magnetic resonance imaging (MRI) of the cervical and thoracic spine was performed. The cervical MRI was clear; however, the thoracic MRI showed passage of the spinal cord through the upper and lower vertebrae at the shortest distance, a tiny herniation of thoracic disc 4–5, and spinal fluid loss anterior to the spinal cord (Fig. 1a, b). These findings were initially considered normal by the general medicine department alone. Although other differential diagnoses were reviewed again, no other diagnoses could be made. However, since the disease onset was triggered by trauma and other diseases had been properly ruled out, the orthopedic surgeon was consulted owing to the suspicion of the possibility of spinal disease, which led him to reach the present findings.

**Fig. 2:**
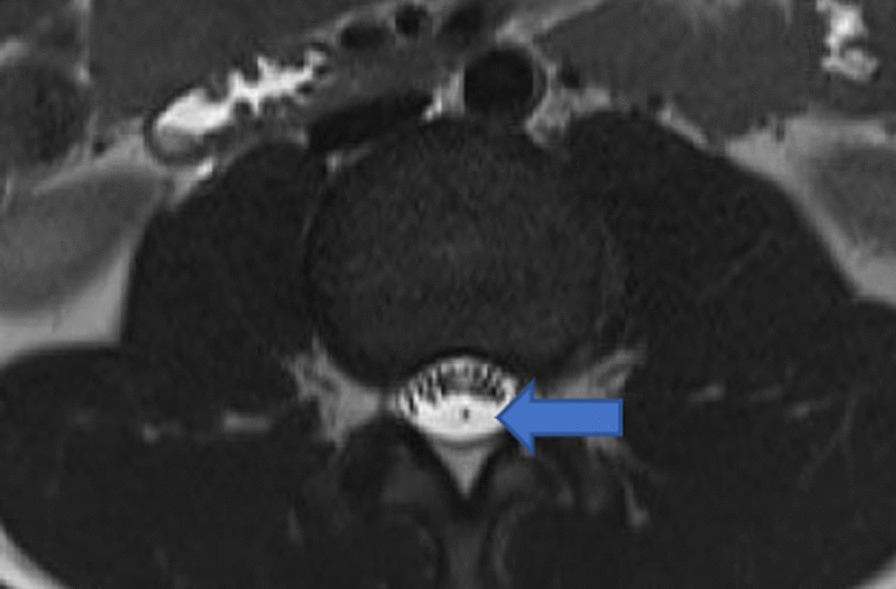
MRI (T2-weighted) of the lumbar spine in the supine position. The filum terminale and cauda equina are separated (arrow)

**Fig. 3:**
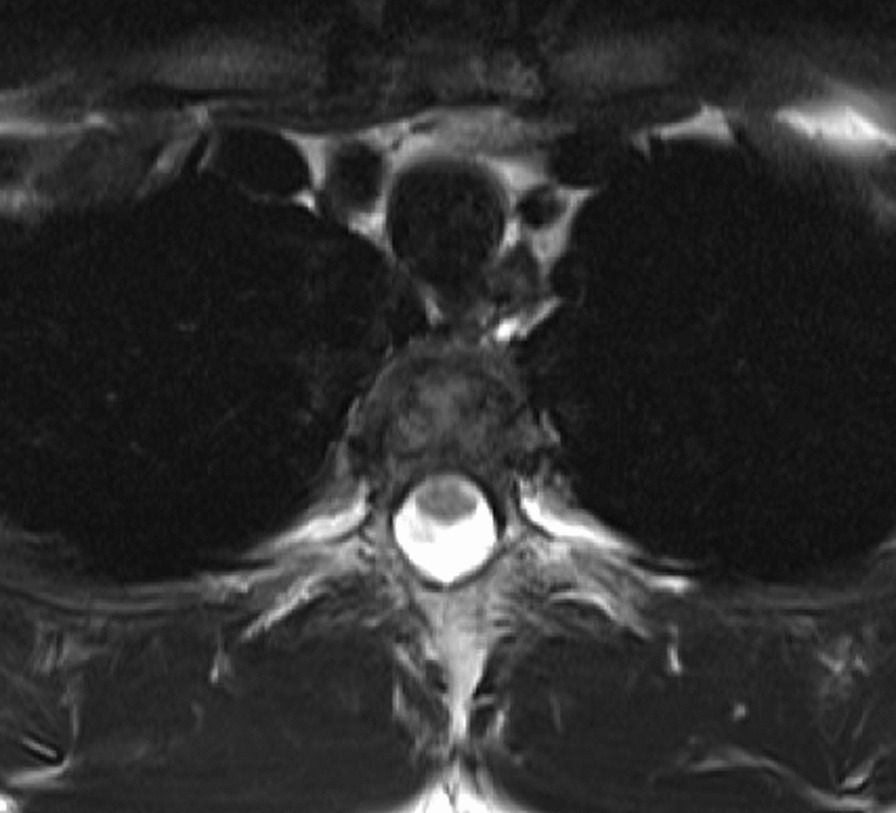
Transverse MRI (T2-weighted) at 4-months post-surgery. MRI findings reveal improved spinal fluid loss in the anterior spinal cord, in the Th4–5 region

These findings suggested a combination of TFT and disc herniation. To diagnose TFT, we performed the finger–floor distance test, which showed a distance of 30 cm between the floor and fingers when bending forward. The TFT-evoked test, induced by forward-bending of the trunk and forward hyperflexion of the neck in a sitting position, was also positive. Subsequently, lumbar MRI was performed in the prone position, revealing a rising sign and leading to a diagnosis of tethered spinal cord syndrome (Fig. 2). At 2 weeks after surgery, the pain almost resolved and the finger-front distance had improved to less than 20 cm. At 2 months after surgery, the pain in the back and upper extremities completely disappeared. Moreover, muscle strength returned to normal. The filum terminale was then surgically removed, and post-surgical follow-up at 7 months confirmed no symptom recurrence. In addition, MRI 4 months after surgery showed increased spinal fluid in the anterior spinal cord (Fig. 3). The fact that the symptoms improved in the absence of any specific treatment for thoracic disc herniation indicated that the symptoms were not caused by thoracic disc herniation alone.

## Discussion

In this case, the combination of TFT and thoracic disc herniation caused repetitive upper extremity symptoms. Several learning points from this case should be noted. TFT has often been acknowledged to be difficult to diagnose because of nonspecific symptoms and unclear imaging findings [6]. As a diagnostic approach, it is important to first identify TFT as a differential, as in this case, and consider the diagnostic criteria developed by Komagata *et al.* [4], consisting of a medical interview and physical examination findings. In addition, a supine MRI scan should be performed, as in the present case, to aid in diagnosis [7].

Previous studies have reported that lower extremity and back pain could occur in almost 100% of adult-onset cases. However, the presence of symptoms in the upper extremities has also been reported, such as in the present case [4, 5, 8]. The onset of upper extremity symptoms is hypothesized to result from traction myelopathy, a condition characterized by central extension to the upper spinal cord, as a result of decreased spinal cord traction reserve [4]. In this case, the symptom specificity in the upper extremities and back was a result of the patient’s original tiny Th4–5 herniation. Moreover, the TFT may have caused external force from sports or other activities in the area of low spinal fluid at the top of the thoracic kyphosis, resulting in direct stimulation of the spinal cord by contact.

A report of six cases with combined TFT/lumbar disc herniation [9] demonstrated that that filum resection was an effective treatment, similar to our observations in this case. However, no symptomatic case with combined TFT/thoracic disc herniation has been reported. Nonetheless, the possibility that TFT has been overlooked because of its rarity cannot be ruled out.

Our patient presented with a post-traumatic sports injury; thus, burners/stingers syndrome was a possible differential. Burners/stingers syndrome is a relatively common disorder in contact sports players that causes unilateral burning pain in the upper extremities and shoulders after injury [10]. Most cases of burners/stingers syndrome resolve within a few minutes, but symptoms may persist for weeks, in rare cases [11]. Therefore, it is important to include TFT in the differential when considering long-lasting burners/stingers syndrome.

This case is also one in which the general medicine physician and orthopedic surgeon collaborated with each other and shared their knowledge to establish a diagnosis that was difficult to make. First, the general medicine physician was able to differentiate various diagnoses as an entry point and narrow down the diagnosis to a musculoskeletal disease. Then, the orthopedic surgeon was able to make a diagnosis on the basis of his own experience in imaging. Expanding knowledge about TFT through this case is important.

## Limitation

Previous reports of TFT in children after surgery reported its recurrence more than 10 years later, requiring a long period of follow-up. However, the short follow-up period of only 7 months in this case could be a limitation [12].

## Conclusion

This case suggests that TFT must be included as a differential diagnosis in cases of back and upper or lower extremity pain with findings that do not match the dermatome. General practitioners, especially those who may treat unexplained symptoms, should be aware of this disease.

## Data Availability

Not available.

## Abbreviations

MRI: Magnetic resonance imaging
TFT: Tight filum terminale
